# Molecular fluctuations in mixed-metal MOF-74: influence of the metal composition

**DOI:** 10.1039/d5ra05357a

**Published:** 2025-08-18

**Authors:** Arda Yildirim, Tabea C. V. Haug, Michael Fröba, Patrick Huber, Andreas Schönhals

**Affiliations:** a Bundesanstalt für Materialforschung und -prüfung (BAM) Unter Den Eichen 87 12205 Berlin Germany Andreas.Schoenhals@bam.de +49 30/8104-73384 +49 30/8104-3384; b Institute for Materials and X-ray Physics, Hamburg University of Technology Denickestr. 17 21073 Hamburg Germany; c Centre for X-ray and Nano Science CXNS, Deutsches Elektronen-Synchrotron DESY Notkestr. 85 22607 Hamburg Germany; d Institute of Inorganic and Applied Chemistry, University of Hamburg Martin-Luther-King-Platz 6 20146 Hamburg Germany; e Institut für Chemie, Technische Universität Berlin Straße des 17. Juni 135 10623 Berlin Germany

## Abstract

A selected series of metal–organic frameworks M-MOF-74 (M = Mg, Co, Ni) and mixed metal MM-MOF-74 (Mg/Co or Mg/Ni) with different compositions of metal atoms have been prepared and further investigated by broadband dielectric spectroscopy (BDS) in a wide temperature range. The dielectric spectra show at least two relaxation processes. Process-A is observed only for the Ni-containing MOFs and is attributed to localized fluctuations of the metal oxide corners. Relaxation processes-B and -C are observed for all prepared MOFs, except that process-B is not observed for Ni-MOF-74. Large-angle fluctuations such as free rotations of the linkers can be excluded due to the structure of MOF-74, but small-angle fluctuations such as torsions are possible. According to numerical simulations carried out for MOF-74, process-B can be attributed to inward and outward fluctuations of the linkers relative to the pore center. Process-C is related to small-angle rotational fluctuations of the linker together with co-rotations of the metal nodes. The latter interpretation is supported by the dependence of the activation energy of the relaxation rate of process-C on the metal composition of the MOFs, which is discussed in terms of the bond lengths between the metal atoms and the linker which decrease in the sequence Mg, Co and Ni.

## Abstract 2

A selected series of metal–organic frameworks M-MOF-74 (M = Mg, Co, Ni) and mixed metal MM-MOF-74 (Mg/Co or Mg/Ni) with different compositions have been prepared and further investigated by broadband dielectric spectroscopy.

## Graphical abstract

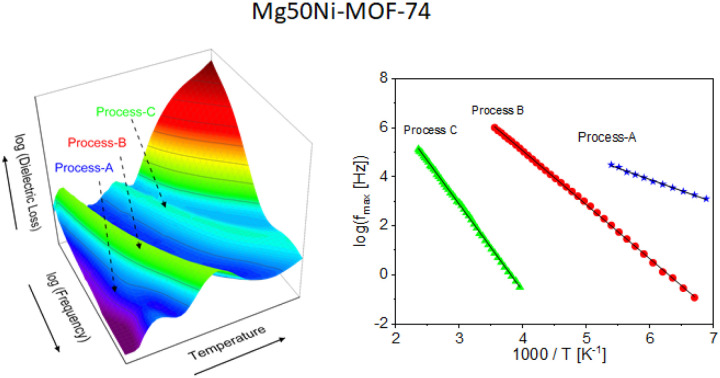

## Introduction

In recent decades, the need for environmental protection, including mitigation of climate change, has increased significantly. Therefore, carbon capture and storage technologies have emerged because of the increasing demand in global energy consumption and the drastically growing global emissions of greenhouse gases, such as CO_2_.^1–3^ In addition to other applications, such as in catalysis, it is not surprising that since the 1990s there has been a strong increase in the synthesis and study of microporous metal–organic frameworks (MOFs).^4^ MOFs are a relatively new class of porous materials that could be used for H_2_ and CH_4_ storage as well as carbon dioxide capture and storage. They could be considered as part of a strategy to address the global challenges mentioned above.

MOFs have extremely high Brunauer–Emmet–Teller (BET) surface area values (up to ∼7800 m^2^ g^−1^) where the corresponding values of the porosity can reach up to 80%.^4–6^ In their structure, metal atoms or clusters of it (secondary building units (SBUs)) are connected by organic linkers. In that way an ordered (crystalline) network with pores, cavities and/or channels is formed. Altering the selection of SBUs employing different metal atoms and/or organic building blocks as organic linkers gives countless design possibilities to synthesize MOF materials having tailored properties and functionalities for targeted specific applications.^5^ Due to their extremely high surface area values and the large design possibilities, MOFs are promising porous materials for a wide range of applications such as gas storage, separation, catalysis besides many others.^6^

Some MOFs have open metal sites (OMS), sometimes also referred to as coordinatively unsaturated sites (CUS), in their structure. This means that not all possible coordination sites of the metals are occupied by linkers. However, during synthesis, these sites are usually filled by solvent molecules. By careful application of heat and/or vacuum after synthesis, if necessary, by first exchanging the synthesis solvent for a less high-boiling solvent, not only the solvent molecules in the pores but also those coordinated at the metal center can be removed. This process is called activation. Typically, OMS are strong adsorption sides for a large number of molecules, which therefore often leads to an increased gas uptake capacity.^7^ MOF-74 are materials which have one of the highest density of OMS.^8,9^ Moreover, because of the possibility to produce MOF-74 at an industrial scale it is considered as a promising material for gas storage and separation processes especially for the gases CH_4_, H_2_ and CO_2_.^10^

MOF-74-type materials typically consists of one type of a divalent metal ion (M^2+^) connected by 2,5-dihydroxyterephthalate linkers which results in a well-defined hexagonally ordered pore network. The pores are channel-like where the 1D metal-oxides SBUs are arranged at the corners of the hexagons with the linkers in-between the corners. These single-metal MOF-74 are denoted as M-MOF-74 where M represents the divalent metal ion, for instance M = Zn^2+^, Mg^2+^, Mn^2+^, Co^2+^*etc.* Unfortunately, MOF-74 materials are relatively unstable towards water. Even humid air can compromise their structural integrity, resulting also in a decreased gas storage capacity.^7,11^ Therefore, different strategies have been developed to improve the water stability and thus maintain the high gas storage capacity.^7,10,11^ One of these strategies is to replace (partially) some metal ions by different metals in the metal nodes in the MOF-74 structure. These materials are denoted as mixed-metal MOFs (MM-MOFs). The intrinsic properties of the metal atoms such as the ionic radius and the electronegativity affect the interaction of the metal nodes with sorbent molecules (*i.e.*, the binding affinity towards guest molecules) and the water stability. This is due to the fact that the electronegativity of the metal atoms affects the length of the M–O bond connecting the metal nodes with the organic linkers, thus the affinity of the metal atoms toward the linkers.^10,14^ It has been proposed that the Lewis acid strength of the metal atoms for adsorption can be attributed to a high electronegativity of the framework oxygen resulting in a higher acidity.^12^ In addition, also a relationship between the stability of the framework and the electronegativity of metal atoms has been suggested.^14^ Furthermore, an inverse relationship between the CO_2_ uptake capacity and the ionic radius of the metal atoms for M-MOF-74 has been reported and discussed considering the electronegativity of the metal centers.^13^

MM-MOF-74 can be synthesized by mixing up to 10 different divalent metals in a single-batch synthesis.^14^ An enhanced water stability as well as higher H_2_ and CO_2_ uptake capacities have been reported for MM-MOF-74 compared to those of M-MOF-74.^15^ As discussed for M-MOFs a major factor in the designing strategy of MM-MOF-74 is also the electronegativity and the radius of the different metal atoms. As discussed above they effect the length of the M–O bond and the affinity of the metal nodes to the linkers.^18^ These factors can be also tailored by the ratio of the different metal atoms. Recently the influence of the metal, the pore size and functionality of a MOF-74 analogue on the structure of confined water was investigated by infrared spectroscopy.^16^

During the last years an increased number of studies of the structure-properties was carried out leading to the development of further design strategies for MOF with OMS.^7^ Despite all these efforts, less is known about the molecular mobility of MOFs. The configuration of the molecules in a MOF structure there possible steric hindrance and molecular interactions might be factors which will influence the molecular mobility. Hitherto, most studies concerning molecular mobility in MOFs employed techniques such as NMR, terahertz spectroscopy as well as computational methods such as DFT calculations and molecular dynamics simulations.^17^ However, Frunza *et al.*^18^ demonstrated that broadband dielectric spectroscopy (BDS) is also a powerful tool to study molecular mobility of MOFs in a broad frequency and temperature range. For MOF-5 several relaxation processes were revealed including small-angle rotational fluctuations of the organic linker. To our best knowledge, until now only scarce investigations concerning the molecular mobility of MOF-74 are known in the literature. For instance, in ref. 19 the influence of the flexibility of two MOF-74 structures on the diffusion of water was discussed employing molecular dynamics simulations. An experimental investigation of the molecular mobility of MOF-74 structures might provide a deeper upstanding of the structure–property relationships. Here, the molecular mobility of a series of single- (M = Mg, Co, and Ni) as well as mixed-metal MOF-74 materials (Mg/Co and Mg/Ni, both in various compositions) is investigated by broadband dielectric spectroscopy. The BET surface area and the porosity of the samples were characterized by N_2_ gas physisorption experiments and thermogravimetric analysis.

## Experimental section

### Materials

Eleven different MOF materials having different metal atoms and varying composition concerning the metal atoms were prepared. Three single-metal MOF-74 (M-MOF-74) having Mg, Ni and Co as metal ions were synthesized. Moreover, eight mixed-metal MOF-74 samples having different Mg/Co and Mg/Ni ratios were prepared. These MM-MOFs are called MgCo-MOF-74 and MgNi-MOF-74. For the synthesis, previously procedures reported elsewhere have been employed.^20,21^ As linker 2,5-dihydroxyterephthalic acid is used. For the synthesis 2,5-dihydroxyterephthalic acid and magnesium nitrate hexahydrate were obtained from Sigma Aldrich GmbH (Germany). Nickel nitrate hexahydrate and magnesium hexahydrate were purchased from ABCR GmbH (Germany). Dimethylformamide and ethanol were delivered by VWR Internation GmbH (Germany). Methanol was obtained By Fisher Scientific GmbH (Germany). All materials were used as obtained.

Exemplary for some samples of the Ni-containing series, the metal composition was investigated using Flame Atomic Absorption Spectroscopy. The corresponding data are provided in the SI. Based on these measurements, it can be concluded that the formulated composition approximately matches the concentration of metal atoms in the synthesized MOF-74s. A similar result is expected for the MgCo-MOF-74 series.

The synthesized materials were further characterized by N_2_ sorption experiments to determine the pore size distribution (PSD), the BET surface area, and the pore volume. Prior to the sorption measurements, the samples were thermally activated by heating them to 393 K and keeping the samples at this temperature for 20 h. For this procedure a MasterPep Degasser Station from Quantachrome was employed. The N_2_-sorption measurements were conducted at 77 K using a Quadrasorb SI-MP from Quantachrome. The examples for sorption and desorption isotherms and for the pore size distributions are given in the SI in Fig. S1 and S2 for different samples. The BET surface value areas were estimated employing the software Quadrawin^22^ in the pressure range *p*/*p*_0_ = 0.0049 … 0.3 (linear range of the sorption/desorption isotherms). The textural properties of the synthesized MOF-74 samples are summarized in Table 1.

**Table 1:** Textual properties of the MOF-74 samples

Sample name	Metal composition [mol%]	Maximum of the PSD [nm]	Surface area [m^2^ g^−1^]	Pore volume [cm^3^ g^−1^]
Mg100-MOF-74	100% Mg	1.27	1420	0.64
Co100-MOF-74	100% Co	1.18	1013	0.59
Ni100-MOF-74	100% Ni	1.14	1471	0.60
Mg90Co-MOF-74	90% Mg, 10% Co	1.22	1590	0.69
Mg70Co-MOF-74	70% Mg, 30% Co	1.18	1166	0.55
Mg50Co-MOF-74	50% Mg, 50% Co	1.18	1200	0.56
Mg25Co-MOF-74	25% Mg, 75% Co	1.07	1091	0.53
Mg90Ni-MOF-74	90% Mg, 10% Ni	1.22	1006	0.51
Mg70Ni-MOF-74	70% Mg, 30% Ni	1.20	1305	0.58
Mg50Ni-MOF-74	50% Mg, 50% Ni	1.22	1243	0.56
Mg25Ni-MOF-74	25% Mg, 75% Ni	1.18	1223	0.57

To confirm the crystal structure of the synthesized MOFs, powder XRD measurements were carried out on the MgNi-MOF-74 and MgCo-MOF-74 series. The X-ray diffraction patterns are provided in the SI, along with a simulated pattern of the Mg-MOF-74 structure (see Fig. S3). The XRD results indicate the formation of proper MOF-74 structures.

### Thermogravimetric analysis (TGA)

The thermal stability of the samples was determined using TGA. The TGA measurements were carried out in the pristine state (as synthesized) employing a Hitachi STA 7200 device. The samples were heated from 303 K to 1273 K with a rate of 10 K min^−1^. Nitrogen was used as a purge gas with a flow rate of 200 ml min^−1^.

Further, isothermal TGA measurements were conducted to determine the time needed for the outgassing procedure, which is necessary to empty the pores and to remove adsorbed water/solvents as well as to optimize the activation of OMS prior to the investigations. These measurements were again carried out by a Hitachi STA 7200 device. The temperature was increased from 303 K to 423 K with a rate of 10 K min^−1^. After the heating step the temperature was kept constant at 423 K for 720 min. During the whole measurement nitrogen was employed as purge gas with a flow rate of 200 ml min^−1^.

**Fig. 1:**
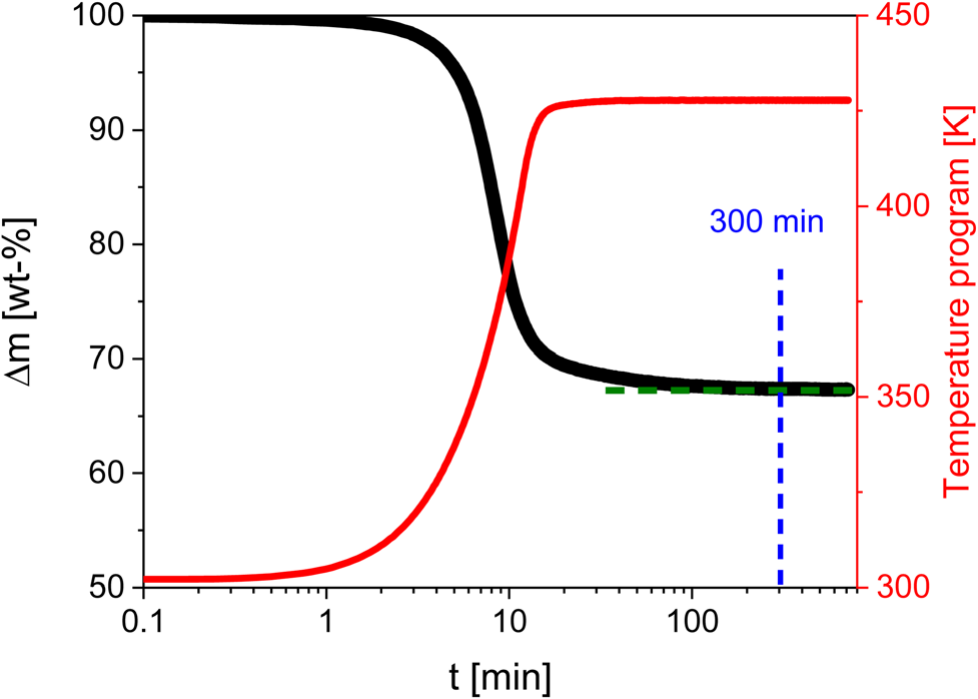
Change of mass (solid black line) for the sample Mg50Ni-MOF-74 and temperature (solid red line) *versus* time. The blue dashed line indicates the determined outgassing time.

Fig. 1 shows the weight loss *versus* time for the sample Mg50Ni-MOF-74 as an example for the determination of the time for outgassing as well as activation of the OMS by isothermal TGA measurements. The TGA curves and the estimated corresponding mass losses for all samples were given in the SI in Fig. S4 and Table S2. The time needed for the outgassing procedure and the activation of OMS is found to be 300 minutes for all samples.

### Broadband dielectric spectroscopy (BDS)

The dielectric properties of the MOF-74 samples were studied by a high-resolution ALPHA analyzer (Novocontrol, Montabaur, Germany) interfaced to a sample cell with an active head. The sample temperature was controlled by a Quatro Novocontrol cryostat with nitrogen as heating/cooling agent (temperature stability better than 0.1 K). More details can be found in ref. 23.

Prior to the BDS investigations, the samples were outgassed in vacuum (10^−4^ mbar) at 423 K for 300 minutes (see above) to empty the pores and to activate the OMS. The samples were transferred under vacuum to an argon-filled glovebox after the outgassing procedure. In the glovebox, the samples were placed into a custom made hermetically sealed dielectric cell. The cell contains stainless steel disk electrodes having an active diameter of 16 mm (parallel plate geometry). The distance between the electrodes was 100 μm, which is maintained by the design of the cell. The details of the dielectric cell are given in ref. 24.

The complex dielectric permittivity ε*(*f*) = *ε*′(*f*) − *iε*′′(*f*) was measured isothermally in the frequency range from 10^−1^ Hz to 10^6^ Hz at temperatures from 133 K to 423 K. Here *ε*′ and *ε*′′ are the real and imaginary (loss) part of the complex dielectric function. 
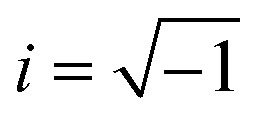
 denotes the imaginary unit and *f* symbolizes the frequency. The samples were first cooled down to 133 K. Then the measurements were started. The temperature program of the measurement consisted of a first heating run from 133 K to 423 K, a first cooling run from 423 K to 133 K and a second heating run from 133 K to 423 K. The temperature increment was 4 K.

## Results and discussion

In the following raw data for selected showcases of each sample group are presented. For M-MOF-74 Mg100-MOF-74, Co100-MOF-74, and Ni100-MOF-74 are selected. As examples for MM-MOF-74 Mg50Co-MOF-74 and Mg50Ni-MOF-74 are chosen. For all other samples, the related information is given in the SI.

**Fig. 2:**
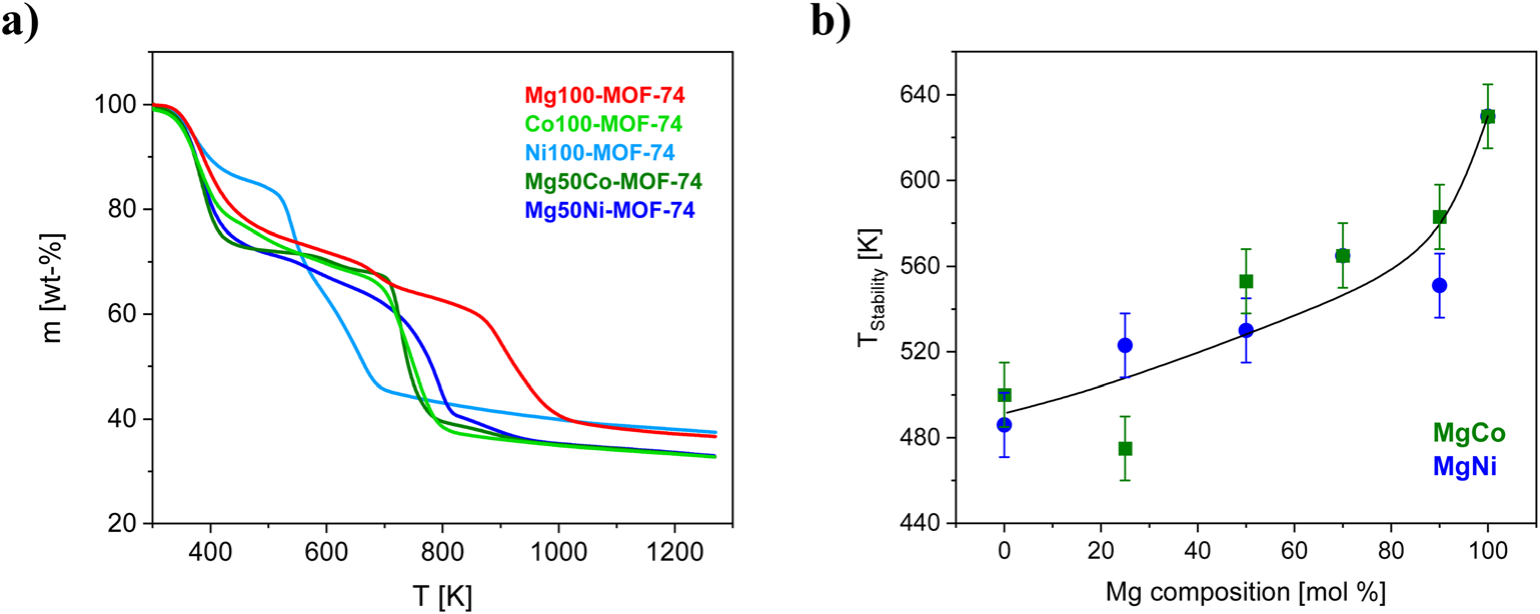
(a) TGA curves for the selected MOF-74s as indicated. (b) *T*_stability_*versus* the Mg concentration as indicated. The solid line indicates the trend in thermal stability with respect to the metal composition.

Fig. 2a shows the thermogravimetric data for the selected MOF-74 samples. For all other samples the TGA curves are given in the SI in Fig. S5. The initial weight loss of *ca.* 15–30% at temperatures between 200 K and 450 K corresponds to the removal of solvent and absorbed water. The weight loss in the temperature range from 450 K to 950 K is assigned to the decomposition of the linker. From the maximum of the derivative mass loss *versus* temperature of the linker decomposition, the linker decomposition temperature (*T*_decomposition_) was estimated. Furthermore, the thermal stability of the MOFs (*T*_stability_) was approximately determined from the deflection point where the mass loss starts to deviate from the baseline after the solvent removal range.

The determined values of *T*_decomposition_ and *T*_stability_ are given in the SI in Table S3. For the M-MOF-74 samples the decomposition temperature of the linker decreases in the order of Mg (*ca.* 904 K) > Co (*ca.* 751 K) > Ni (*ca.* 658 K). For the series of MgCo-MOF-74 samples, except for the sample Mg25Co-MOF-74 the decomposition temperatures increase with increasing Mg content towards to the decomposition temperature for Mg100-MOF-74.

Also, the decomposition temperatures for MgNi-MOF-74s and MgCo-MOF-74 increase also with increasing amount of Mg. Furthermore, the thermal stability of the MOF-74s follows the same order as *T*_decomposition_ and increases with the concentration of Mg (see Fig. 2b). The dependence of *T*_stability_ on the Mg concentration seems to be intendent of the co-metal. This behavior is expected since the linker decomposition temperature is often taken as indicator for the thermal stability of MOFs. These findings are also in agreement with the literature data for both the M-MOF-74 and the MM-MOF-74 materials.^25,26^ However, the absolute values of *T*_decomposition_ and *T*_stability_ obtained here differ a bit from the data reported in the literature because of different heating rates and the purge gas used in the TGA measurements as well as differences in the synthesis. Furthermore, it can be speculated that the intrinsic properties of the metals in the structure of the MOF-74s such as ionic radius, bonds lengths and electronegativity might have an influence on their thermal stability. However, to make more precise statements, further studies on thermal stability of M-MOF-74 and MM-MOF-74 having different metals atoms such Zn, Mn, Fe and Cd are required.

**Fig. 3:**
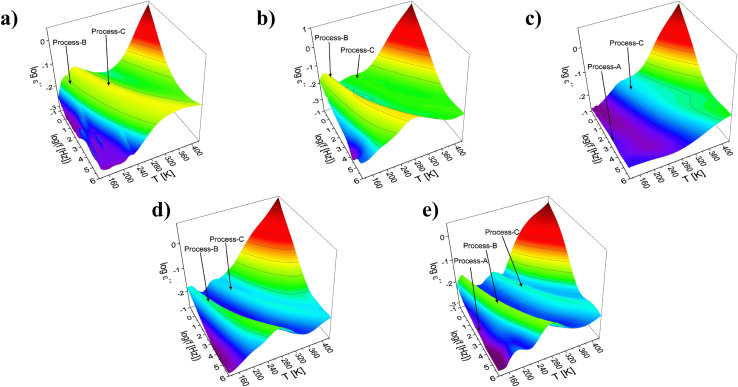
Dielectric loss *ε*′′ as function of frequency and temperature for (a) Mg100-MOF-74, (b) Co100-MOF-74, (c) Ni100-MOF-74, (d) Mg50Co-MOF-74 and (e) Mg50Ni-MOF-74 in 3D representations for heating cycle. Arrows indicate the dielectric relaxation processes.

Examples for the dielectric investigations are given in Fig. 3 as 3D representations of the dielectric loss as function of frequency and temperature for the selected samples. For the other studied materials, the 3D representations of the dielectric loss are depicted in the SI in Fig. S6. In general, at least two dielectrically active relaxation processes were observed as peaks in the dielectric loss for all MOF-74 samples considered here. The detected relaxation processes are named as process-A, -B, and -C. Process-A appears as a high frequency shoulder of process-B and was observed only for the Ni-containing MOF-74 samples. Process-B and -C were in principle detected for all MOF-74 materials except for Ni100-MOF-74 where process-B is not observed. In addition to the relaxation processes, a conductivity contribution together with parasitic electrode polarization is observed at low frequencies and higher temperatures as a strong increase of the dielectric loss with decreasing frequency.

To analyze dielectric data the model function of Havriliak and Negami (HN-function) is fitted to the data.^27^ The HN-function describes both a symmetric and asymmetric broadening of a relaxation peak. The HN-function reads1
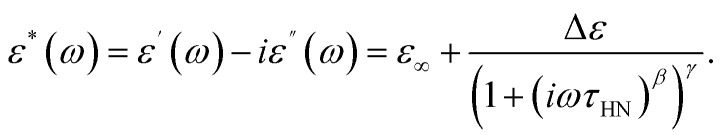


The fractional shape parameters *β* and *γ* (0 < *β*; β*γ ≤ 1) describe the functional shape of the relaxation spectrum (symmetric and asymmetric broadening) compared to the Debye one.Δ*ε* denotes the dielectric strength whereas *τ*_HN_ is a relaxation time corresponding to the frequency at maximal dielectric loss *f*_max_ (relaxation rate) by^28^2
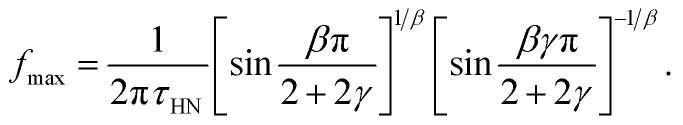


If more than one relaxation process is observed in the considered frequency window a sum of HN-functions is fitted to the data. Contributions related to conductivity were treated in a conventional way by including the term 
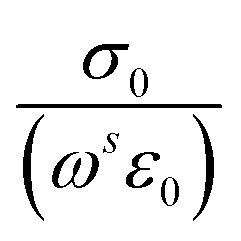
 to the loss part of the HN-function. Here, *σ*_0_ is related to the DC conductivity and *s* is a parameter describing non-ohmic effects in the conductivity (0 < *s*< =1). *s* = holds for pure ohmic conductivity. For further details see ref. 28.

**Fig. 4:**
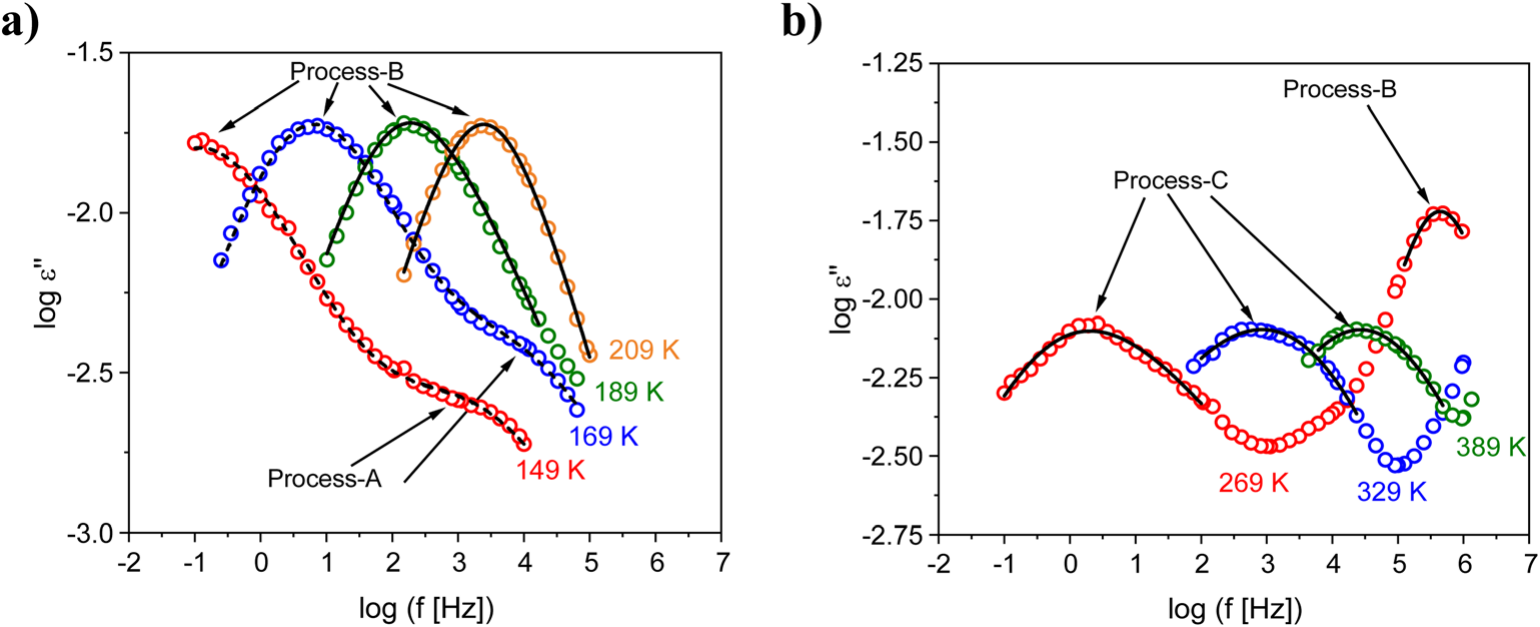
Dielectric loss *ε*′′ *versus* frequency for the indicated temperatures: (a) process-A and -B as well as (b) for process-B and -C for the sample Mg50Ni-MOF-74. Solid lines are fits of a single HN-function to the data whereas dashed lines are fits of the sum of two HN-functions to the data.

**Fig. 5:**
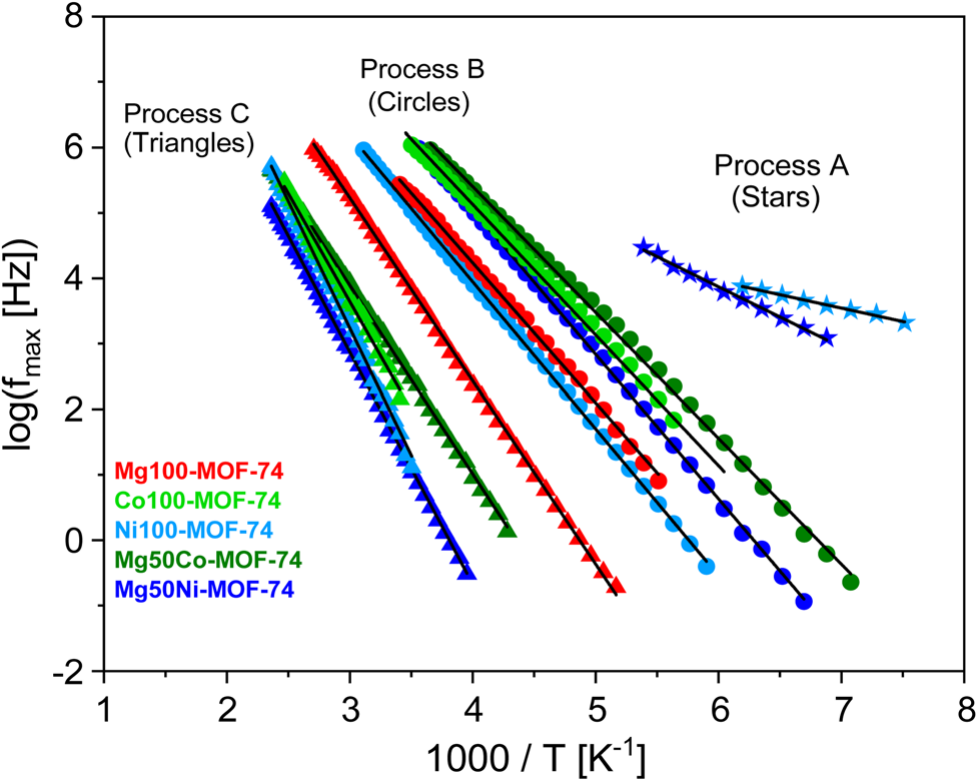
Relaxation map (Arrhenius diagram) for the selected MOF-74 materials. Different symbols indicate the different processes as indicated. Each color represents a specific sample as indicated on the left bottom. Solid lines are fits of the Arrhenius equation to the data.

Examples for the fits of the HN-function to the data are given in Fig. 4 for the sample Mg50Ni-MOF-74. From the fits the relaxation rate *f*_max_ is obtained. For the selected showcases, the estimated relaxation rates for the different processes are plotted *versus* inverse temperature in the Arrhenius diagram (relaxation map, Fig. 5).

For each process and all samples, the temperature dependence of the relaxation rates can be described by the Arrhenius equation which reads3
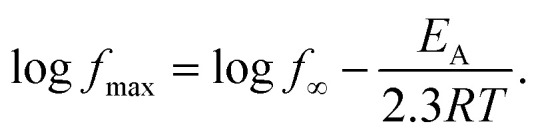
Here *f*_∞_ is the pre-exponential factor, *E*_A_ symbolizes the activation energy, and *R* is the universal gas constant. The estimated activation energies and the pre-exponential factors for all detected processes for all samples are collected in the SI in Table S4. For process-A of the sample Mg50Ni-MOF-74, an activation energy of 18 kJ mol^−1^ and a value of the pre-exponential factor log(*f*_∞_ [Hz]) = 9.4 were estimated. The low value of the activation energy indicates localized molecular fluctuation. For MOF-5 and ZIF-8 such a localized process was also reported and assigned to fluctuations of the corners of the cages for MOF-5 and ZIF-8.^18,29^ Similar to the structures of MOF-5 and ZIF-8, metal-oxide clusters form also the corners of the pores for MOF-74 s. One has to note that for MOF-74 the SBUs can be considered as a 1D rod-like structure where that of ZIF-8 and MOF-5 are 0D clusters. Although the structures of the SBUs of MOF-5/ZIF-8 are different compared to that of MOF-74 as well as also the metal atoms are different localized fluctuations of the corners formed by the metal-oxide clusters are suggested as molecular origin for process-A. The fluctuations of metal nodes located at the corner of the pores are also discussed in ref. 30 and 31 for MOF-74 from a theoretical point of view. Moreover, process-A is observed only for the Ni-containing MOF-74 samples as a shoulder of the process-B. This makes the analysis and thus the assignment of the process-A relatively difficult. Therefore, this process will be not further discussed for this material.

The activation energies for the process-B and -C were estimated to be in the range of 36–43 kJ mol^−1^ and 54–77 kJ mol^−1^, respectively. The values are in agreement with the activation barriers for the rotational fluctuations of the organic linkers for a some of MOFs like MOF-5 and UiO-66.^18,32^ Furthermore, the pre-exponential factors determined for these processes agree with literature values of the pre-exponential factor for the motions in the organic linkers for instance the fluctuations of the phenyl rings.^18^ For MOF-5 such an assignment is in agreement with NMR measurements.^35^ It should be explicitely noted that such fluctuations does not mean 180° rotations. These molecular motions should be considered rather as small angle fluctuations. Therefore, it might be concluded that the molecular origin of these processes is related to the organic linkers of the MOF-74 materials although the linkers and how they are incorporated into the network are different. This assignment is further supported by simulations.^31^ Moreover it was also shown that the fluctuations of the linkers can affect the properties of MOFs like the gas uptake and diffusion.^33^

Process-B and -C are observed as distinctive peaks in the relaxation spectra (see Fig. 3). Therefore, they should have different molecular origins. The higher activation energies estimated for process-C indicates stronger restrictions to the fluctuations compared to that involved in process-B should be considered for the discussions.

Large angle fluctuations like free 90 or 180° jumps *etc.* are excluded as molecular origin for fluctuations by the special mode of incorporation of the linkers into the structure of MOF-74 as discussed above. Kamencek *et al.*^30^ investigated the origin of the anisotropic thermal expansion of MOF-74 using simulations and temperature dependent X-ray diffraction. They reported two possible kinds of molecular fluctuations of the linkers in the range of THz frequencies. One process is related to inward and outward fluctuations of the linker relative to the pore center. According to the simulations it is induced by the counter-rotation of the corners. This process can be considered as a torsional fluctuation of the linkers. Small angle rotational fluctuations of the linker together with co-rotations of the metal nodes is considered as the second process. According to the simulations the former process requires less stretching of the linker where the linker fluctuates mainly as a rigid unit, the latter process leads to a change of the pore wall structure and requires thus a stretching of the linker. Although both processes discussed by Kamencek *et al.*^30^ are observed in the THz frequency range the same picture can be used to discuss the dielectric spectra at lower frequencies.

**Fig. 6:**
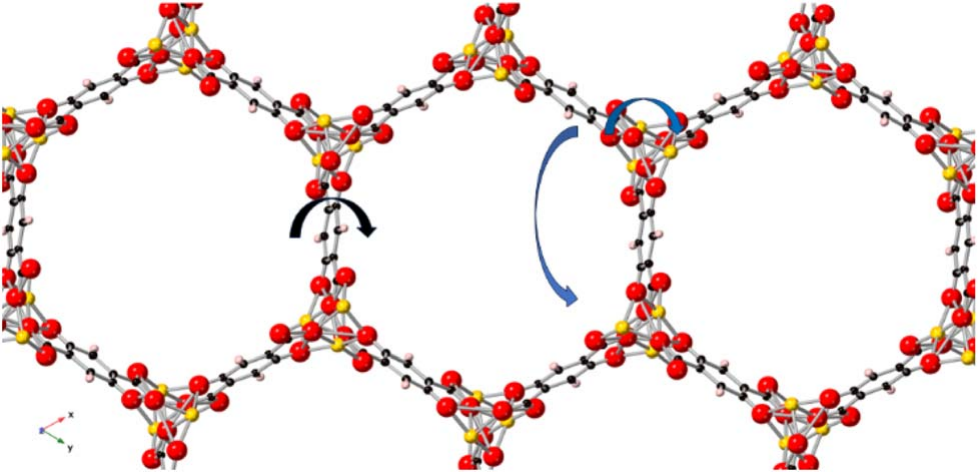
Top view of the Mg100-MOF-74 structure: red – oxygen, yellow-magnesium, violet – hydrogen and back – carbon. The structure was created by the software Crystal Maker. The arrows indicate the suggested relaxation processes: blue – process-B; black – process-C.

The spectral shape of the process-B is narrower than that of the process-C and resembles that of a Debye-like process (see Fig. 4b). A relaxation process with a narrow spectrum indicates a narrow distribution of relaxation times which points to the conclusion that this process takes place in a well-defined structure. Fig. 6 depicts the structure of Mg100-MOF-74 as example for the investigated MOF-74 series. In the structure of MOF-74 the edges of the pores are organized in a hexagonal array, which form a well-defined pore structure. The inward and outward fluctuations of the linker in MOF-74 will take place in this well-ordered pore structure compared to the rotational fluctuations of the linker on the pore edges. Therefore, process-B might be assigned to the inward and outward fluctuations of the linker. An illustration of this process is given in Fig. 6. Likely these fluctuations are mainly due to that of the phenyl ring. The determined value of log(*f*_∞_ [Hz]) values for process-B are found in the lower THz compared to that of process-C which agrees with the reported inward and outward motion of the linker at lower THz frequency. Moreover, the estimated values for the activation energies of process-B are comparable to that of the rotational fluctuations of the phenyl rings in polycarbonate.^34^

The process-C will be assigned to small-angle rotational fluctuations of the linker. A schematic of process-C is given in Fig. 6. These linker fluctuations are subjected to a higher restriction compared to the inward and outward motion of the linker since a such a rotational fluctuation will lead to a dynamic change in the pore wall structure as discussed above.^30^ This might be the reason for the higher activation energies found for process-C compared to that for process-B.

Only a scarce number of investigations regarding the molecular dynamics of MOFs are available in the literature. Nevertheless, a comparison of the small angle rotational fluctuations of MOFs discussed in the literature might shed some light on the understanding of the molecular dynamics of MOF-74. Gould *et al.*^35^ revealed the rotational fluctuations of the phenylene rings in MOF-5 by NMR. Therefore, these NMR data are added to the Arrhenius diagram given in Fig. S7 in the SI for the comparison. The Arrhenius diagram exhibits that the process-C of Mg100-MOF-74 and the NMR data can be well described by a common Arrhenius dependency. This agreement supports further the assignment of process-C to small angle rotational fluctuations of the linker. However, this agreement between Mg100-MOF-74 and the NMR data of MOF-5 is not observed for the other MOF-74 samples. This indicates that employing different metals and different compositions of these metal atoms in the structure of MOF-74 have a significant effect on the small angle rotational fluctuation of the organic linker.

**Fig. 7:**
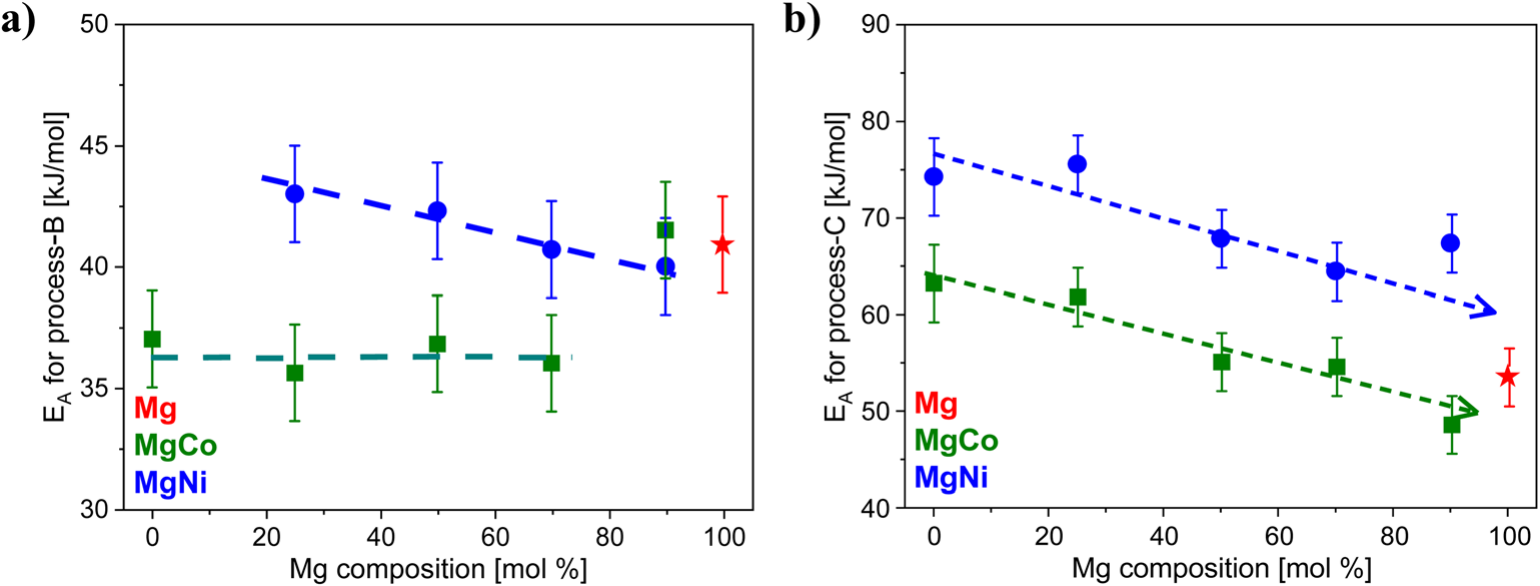
Dependence of activation energies on Mg concentration for (a) process-B and (b) process-C. Dashed blue and black lines are the guides to the eye.

**Fig. 8:**
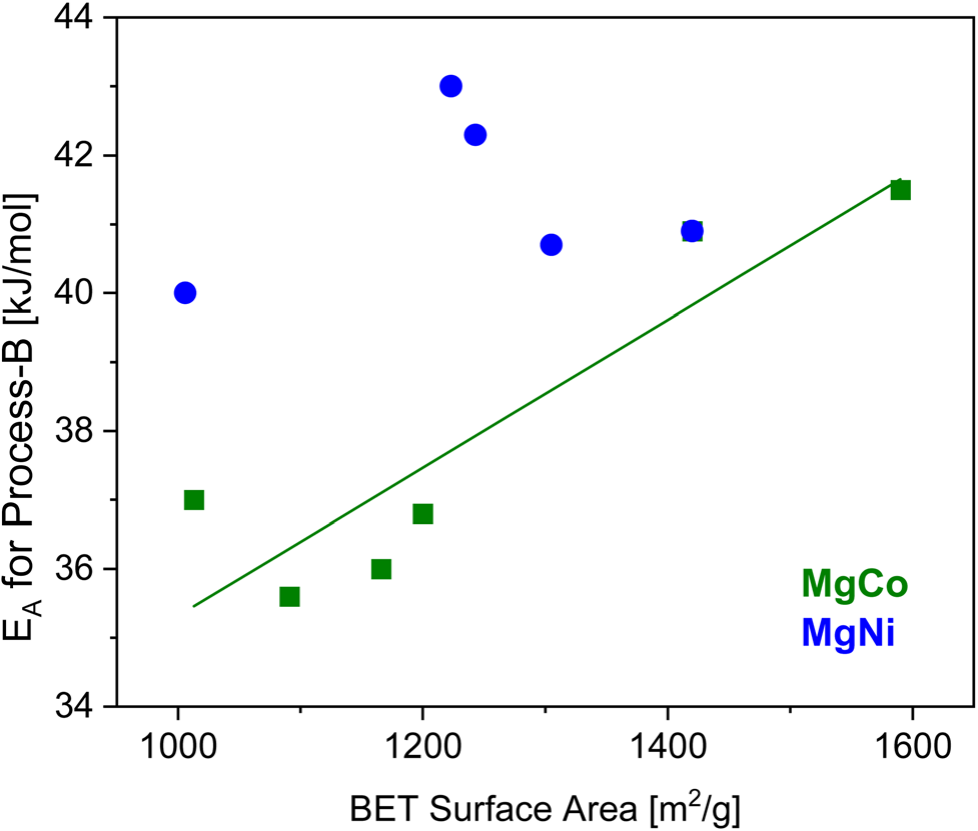
Activation energy *versus* the BET surface area as indicated. The line is a linear regression to the data of MgCo-MOF-74. The regression coefficient *R*^2^ > 0.8.

To evaluate the possible effects of the metal composition on the molecular dynamics of the linker, the activation energies for process-B and -C are plotted *versus* the molar concentration of Mg (see Fig. 7). On the one hand, the activation energies for process-B are slightly lower for the MgCo-MOF-74 series compared to that of the MgNi-MOF-74 ones. For the MgCo-MOF-74 series the activation energy is independent of the Mg content in the samples until a Mg content of 70 mol% (see Fig. 6a). For higher concentrations of Mg, the activation energy for process-B jump to the value obtained for Mg100-MOF-74. At the same concentration also a jump of the value BET surface area to higher values is also observed for the MgCo-MOF-74 series. It might be discussed that there is a correlation between the in and out fluctuation of the linker (process B). This discussion might be supported by the correlation of the BET surface area value and the activation energy of process-B (see Fig. 8). For the MgNi-MOF-74 series the activation energy of the process-B decreases slightly towards the activation energy found for Mg100-MOF-74. For this series no correlation is found of the BET surface area value and the activation energy of process-B (see Fig. 8). This might be due to the larger binding length found for Ni containing system. Nevertheless, the differences between the activation energies for process-B for both systems and all concentrations of the metal atoms are relatively small. Therefore, it is concluded that the inward and outward fluctuations of the linker depend only weakly on the metal composition which supports the assignment. This is probably due to the fact that the linker fluctuates as a rigid unit which is less dependent of the metals in the SBUs. For process-C the activation energies decrease for both the Ni- and the Co-containing MOF-74 samples with increasing Mg concentration towards to the activation energy for Mg100-MOF-74 (see Fig. 6b). Moreover, the activation energies are higher for the Ni-containing MOF-74 samples than the activation energies for Co-containing ones. For process-C, these dependencies point to the influence of the composition of the metals on the energy barrier for the small angle or torsional rotational fluctuations of the organic linker. This supports the assignment of process-C as this process involves a dynamical change of the pore structure. In this context it is worth noting that the bond lengths between the metal atoms and the linker decrease in the sequence Mg, Co, and Ni. The shorter bonds length for the MM-MOF-74 with Ni in comparison to the Co containing ones lead to stiffer linkers leading to the higher activation for MgNi-MOF-74 for both processes.

The influence of the metal cluster on the structure and the properties of MOFs are often discussed by considering the electronegativity or Lewis acidity strength of the metal atom in MOF-74.^36^ The electronegativity values of Mg, Co and Ni atoms are ranked as Ni (1.91) > Co (1.88) > Mg (1.31) according to the Pauling scale.^37^ The dependencies given in Fig. 6b for process-C are in qualitative agreement with the average electronegativity (considering the ratio of the metals) of the metals in the MOF-74 samples. It might be discussed that the metal atoms with a higher electronegativity shifts the negative charge gravity of the oxygens of the linker to the M–O bond (where M is Mg, Co, or Ni) in MOF-74. A higher average electronegativity results in a stiffer linker that leads to a higher restriction of its the small angle rotational fluctuations of the organic linker, which requires a dynamical change of the pore network. Furthermore, computational, and experimental investigations showed that the M–O bond length increases as the molar Mg concentration increases in MgNi-MOF-74 materials. Nevertheless, more investigations are required to better understand the effect of mixing metals at different ratios on the rotational fluctuations of the organic linker.

## Conclusions

The manuscript reports an investigation of the molecular mobility of mixed-metal MOFs of the series MOF-74 employing broadband dielectric spectroscopy. For each sample their dielectric spectra show at least two relaxation processes besides conductivity related contributions. Process-A is only observed for the Ni containing MOFs as a shoulder of the process-B. This process is assigned to fluctuations of metal nodes at the edges of the pores. The processes- B and –C are observed for all prepared MOF materials with the exception that process-B is not observed for Ni100-MOF-74. These assignments agree with literature results obtained for single MOF-74 materials by simulations and FTIR measurements. One must note that due to the structure of MOF-74 materials large angle rotational fluctuations of the linkers are not possible for MOF-74. However, this is not true for small angular fluctuations such as torsions. Molecular simulation evidence that two types of molecular fluctuations are possible in a MOF-74 structure on the terahertz frequency scale. The first process is related to inward and outward fluctuations of the linker with respect to the pore center. Such a process is assigned as molecular origin also of process-B. The activation energy of process-B depends only weakly on the composition of the metals in the mixed-metal MOF-74. Therefore, it is concluded that the linker fluctuates as a rigid unit which supports the assignment of process B. The second process discussed in the simulation are small angle rotational fluctuations of the linker together with co-rotations of the metal nodes. Such a process is considered as molecular origin for process-C. As the metal modes are involved in this process a dynamical change of the pore network is required, the activation energy depends on the on the metal composition of the MOF-74 material. For both series the activation energy decreases with decreasing Ni and Co concentration to that value found for Mg-MOF-74. This result is discussed by the decreasing electronegativity with increasing Mg content because the M–O bond becomes weaker with decreasing electronegativity. This means that the stretching of the M–O bond is less restricted when the Mg concentration increases. The activation energy of the process-C is higher for the NiMg-MOF-74 compared to that of the Co containing one. This behavior can be understood by the higher electronegativity of Ni. The obtained results should be confirmed by investigations on further mixed-metal MOFs.

## Conflicts of interest

The authors confirm that they have no conflicting interests.

## Supplementary Material

RA-015-D5RA05357A-s001

## Data Availability

Data will be available from the corresponding author on a reasonable request. Nitrogen physisorption isotherms (77 K) and pore size distribution; TGA thermograms; estimated stability and decomposition temperatures. Dielectric spectra as dielectric loss *versus* frequency and temperature; table of the activation energies; relaxation map. See DOI: https://doi.org/10.1039/d5ra05357a.
