# Supplementary material for: Molecular fluctuations in mixed-metal MOF-74: influence of the metal composition
Source: RSC Adv. 2025 Aug 18;15(35):29109–18. doi: 10.1039/d5ra05357a (PMC12376766; doi:10.1039/d5ra05357a)
Supplement: RA-015-D5RA05357A-s001 [file RA-015-D5RA05357A-s001.pdf]

Supplementary Information (SI) for RSC Advances.
This journal is © The Royal Society of Chemistry 2025

Supporting Information

Molecular Fluctuations in Mixed-Metal MOF-74: Influence of the Metal Composition

Arda Yildirim[1,2,3,§], Tabea Haug[4], Michael Fröba[4], Patrick Huber[2,3], and Andreas Schönhals[1,5,*]

[1]Bundesanstalt für Materialforschung und -prüfung (BAM), Unter den Eichen 87, 12205 Berlin, Germany

[2]Institute for Materials and X-ray Physics, Hamburg University of Technology, Denickestr. 17, 21073 Hamburg, Germany

[3]Centre for X-ray and Nano Science CXNS, Deutsches Elektronen-Synchrotron DESY, Notkestr. 85, 22607 Hamburg, Germany

[4]Institute of Inorganic and Applied Chemistry, University of Hamburg, Martin-Luther-King-Platz 6, 20146 Hamburg, Germany

[5]Institut für Chemie, Technische Universität Berlin, Straße des 17. Juni 135, 10623 Berlin, Germany

[§]Current address: One.five GmbH, Reichkanzlerstraße 2a, 22609 Hamburg, Germany

[*]CORRESPONDING AUTHOR: A. Schönhals, BAM Bundesanstalt für Materialforschung und -prüfung (Department Materials Chemistry), Unter den Eichen 87, 12205 Berlin, Germany; Tel. +49 30 / 8104-3384; Fax: +49 30 / 8104-73384; Email: Andreas.Schoenhals@bam.de

Flame Atomic Absorption Spectroscopy

The Flame Atomic Absorption measurements were carried out with the device Solaar S Series (Thermo). For the Flame Atomic Absorption Spectroscopy for measurements one and two the sample was digested in 1:1 mixture of HNO_3 and $HClO_4$. For measurement three the sample was digested in HNO_3.

Table S1:

Material	Measurement	Composition Mg [%]	Composition Ni [%]
Mg/Ni(9:1)	1	13.7	5.0
	2	13.7	4.9
	3	13.9	4.9
	Theoretical	17.5	4.7
Sum formula: $Mg_{1.7}Ni_{0.3}$(dobdc), actual ratio: 5.7:1, molar mas: 253.1 g/mol			
Mg/Ni(7:3)	1	9.4	14.2
	2	9.5	14.7
	3	9.2	14.2
	Theoretical	12.9	13.4
Sum formula: $Mg_{1.2}Ni_{0.8}$(dobdc), actual ratio: 3:2, molar mas: 270.3 g/mol			
Mg/Ni(1:1)	1	5.5	21.5
	2	5.3	21.6
	3	5.4	22
	Theoretical	8.8	21.2
Sum formula: $Mg_{0.75}Ni_{1.25}$(dobdc), actual ratio: 3:5, molar mas: 285.7 g/mol			

Nitrogen Adsorption Measurements

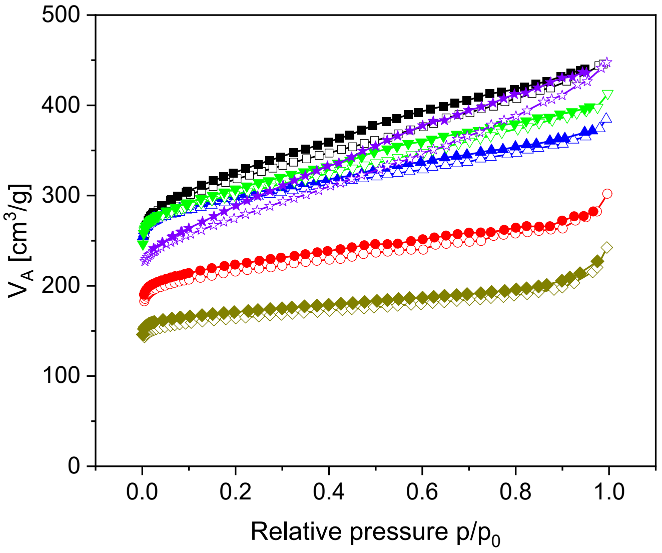

Figure S1a. Nitrogen physisorption isotherms (77 K) for the system MgNi-MOF-74: black squares – 100 % Mg, red circles – 10% Ni, blue up sided triangles – 30 Ni, green down sided triangles – 50 % Ni, violet asterisk – 75% Ni and yellow diamonds – 100% Ni. Open symbols – sorption, filled symbols – desorption.

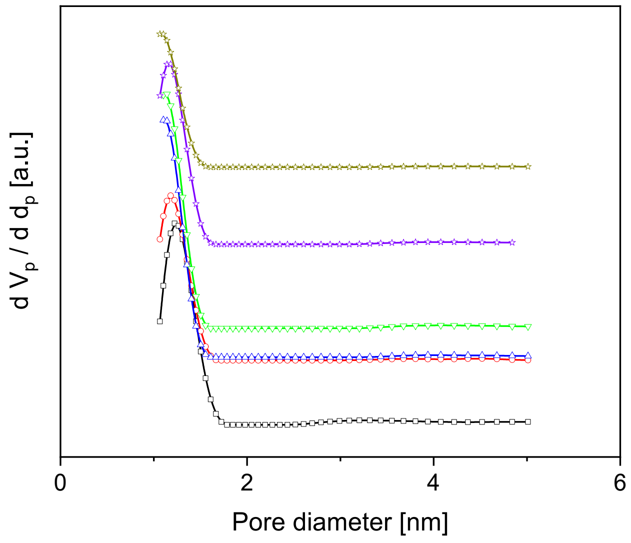

Figure S1b. Pore size distribution for the system MgNi-MOF-74: black squares – 100 % Mg, red circles – 10% Ni, blue up sided triangles – 30 Ni, green down sided triangles – 50 % Ni, violet asterisk – 75% Ni and yellow diamonds – 100% Ni. The curves are shifted along the y-scale for sake of clearness.

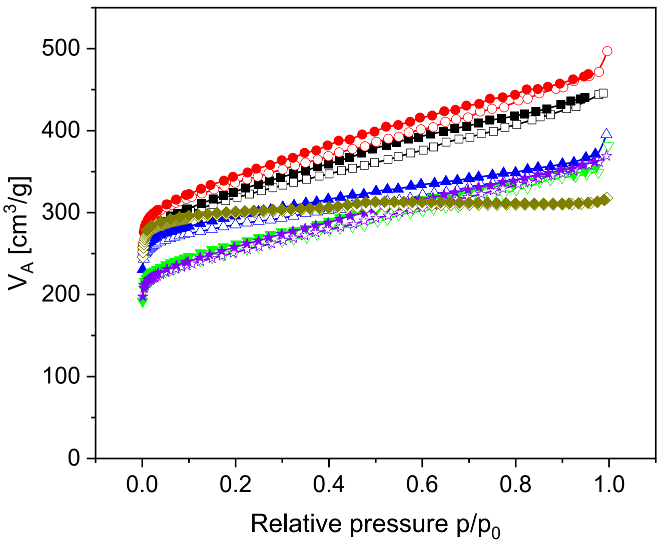

Figure S2a. Nitrogen physisorption isotherms (77 K) for the system MgCo-MOF-74: black squares – 100 % Mg, red circles – 10% Co, blue up sided triangles – 30 Co, green down sided triangles – 50 % Co, violet asterisk – 75% Co and yellow diamonds – 100% Co. Open symbols – sorption, filled symbols – desorption.

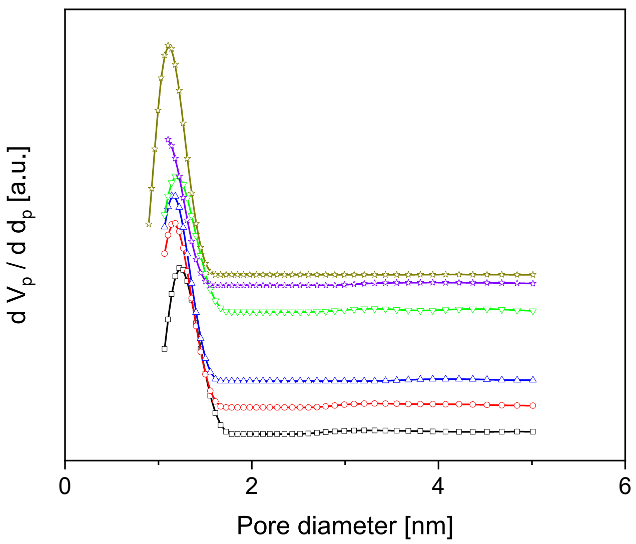

Figure S2b. Pore size distribution for the system MgNi-MOF-74: black squares – 100 % Mg, red circles – 10% Co, blue up sided triangles – 30 Co, green down sided triangles – 50 % Co, violet asterisk – 75% Co and yellow diamonds – 100% Co. The curves are shifted along the y-scale for sake of clearness.

XRD Measurements

The powder XRD measurements were carried out with X'Pert Pro PW3040/60 (PANalytical) device in θ/θ (Bragg-Brentano) geometry employing CuK_{α} radiation (45 kV, 40 mA; counting time: 74 s; step size: 0.013° (2θ)). Data analysis was carried out with the software X'Pert High-Score Plus.

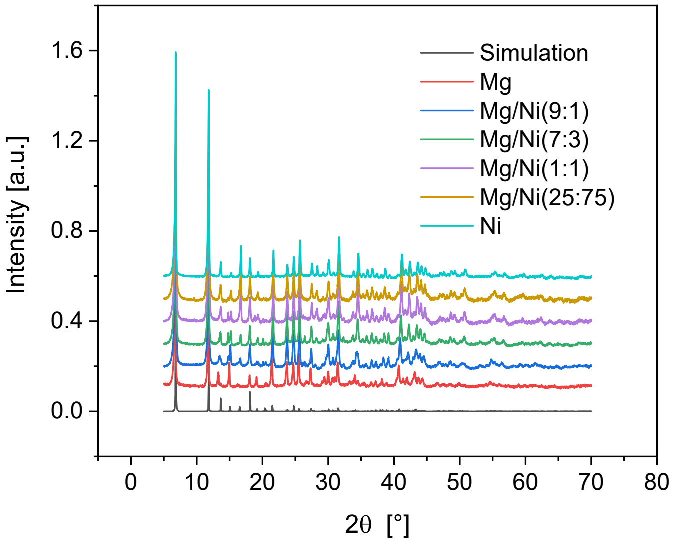

Figure S3a. XRD for the MgNi-MOF-74 series as indicated.

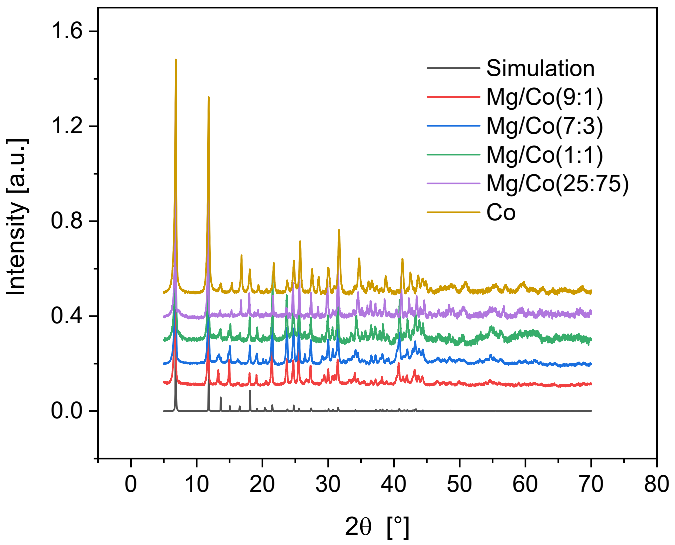

Figure S3b. XRD for the MgCo-MOF-74 series as indicated.

TGA Measurements

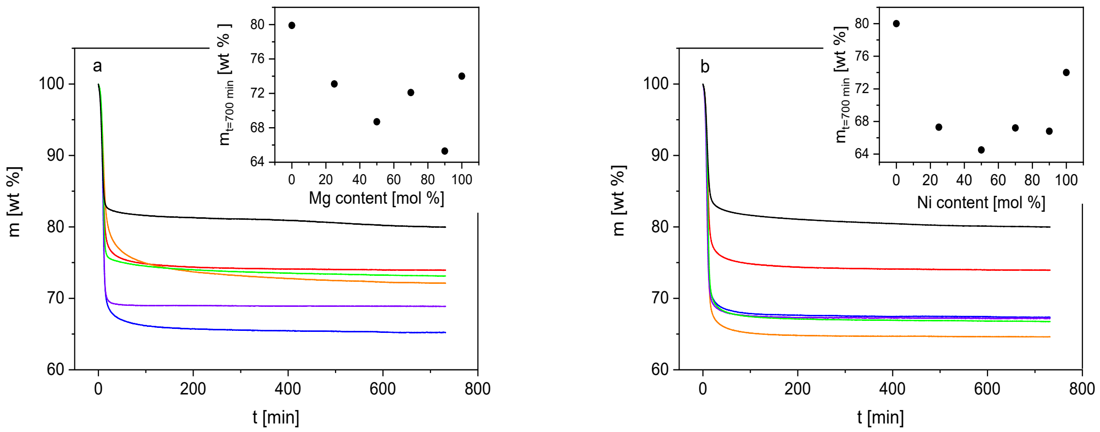

Figure S4. Mass change versus time at a temperature of 420 K. a MgCo-MOF-74: black 0 mol % Mg; green 25 mol % Mg; violet 50 mol % Mg; orange 70 mol % Mg. blue 90 mol % Mg; red 100 mol % MG. The inset gives the weight loss at t=700 min versus the Mg content. b MgNi-MOF-74: black 0 mol % Mg; green 25 mol % Mg; violet 50 mol % Mg; orange 70 mol % Mg. blue 90 mol % Mg; red 100 mol % Mg. The inset gives the weight loss at t=700 min versus the Mg content.

Table S2. The determined the outgassing time and total mass loss

Material	Temperature [K]	Total Mass Loss [%]	Determined Outgassing Time [min]
Mg100-MOF-74	423	26	300
Co100-MOF-74	423	20	300
Ni100-MOF-74	423	20	300
Mg90Co-MOF-74	423	35	300
Mg70Co-MOF-74	423	28	300
Mg50Co-MOF-74	423	31	300
Mg25Co-MOF-74	423	27	300
Mg90Ni-MOF-74	423	33	300
Mg70Ni-MOF-74	423	35	300
Mg50Ni-MOF-74	423	33	300
Mg25Ni-MOF-74	423	33	300

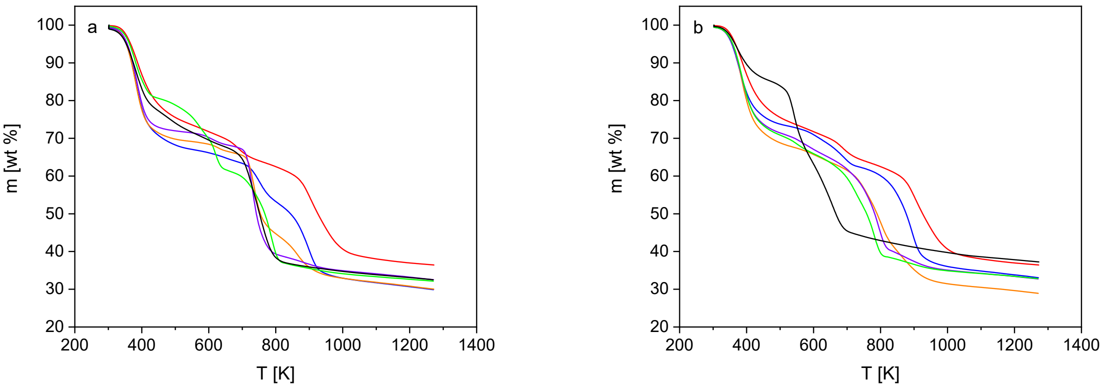

Figure 5. Mass change versus temperature. a - MgCo-MOF-74: black 0 mol % Mg; green 25 mol % Mg; violet 50 mol % Mg; orange 70 mol % Mg; blue 90 mol % Mg; red 100 mol % Mg. b - MgNi-MOF-74: black 0 mol % Mg; green 25 mol % Mg; violet 50 mol % Mg; orange 70 mol % Mg. blue 90 mol % Mg; red 100 mol % Mg.

Table S3. Decomposition temperature of the linker and thermal stability of the MOFs

Name	$T_{decomposition}$	$T_{stability}$
Mg100-MOF-74	904 K	630 K
Co100-MOF-74	751 K	500 K
Ni100-MOF-74	658 K	486 K
Mg90Co-MOF-74	900 K	583 K
Mg70Co-MOF-74	735 K	565 K
Mg50Co-MOF-74	730 K	553 K
Mg25Co-MOF-74	787 K	475 K
Mg90Ni-MOF-74	895 K	551 K
Mg70Ni-MOF-74	801 K	565 K
Mg50Ni-MOF-74	797 K	530 K
Mg25Ni-MOF-74	783 K	523 K

Dielectric Measurements

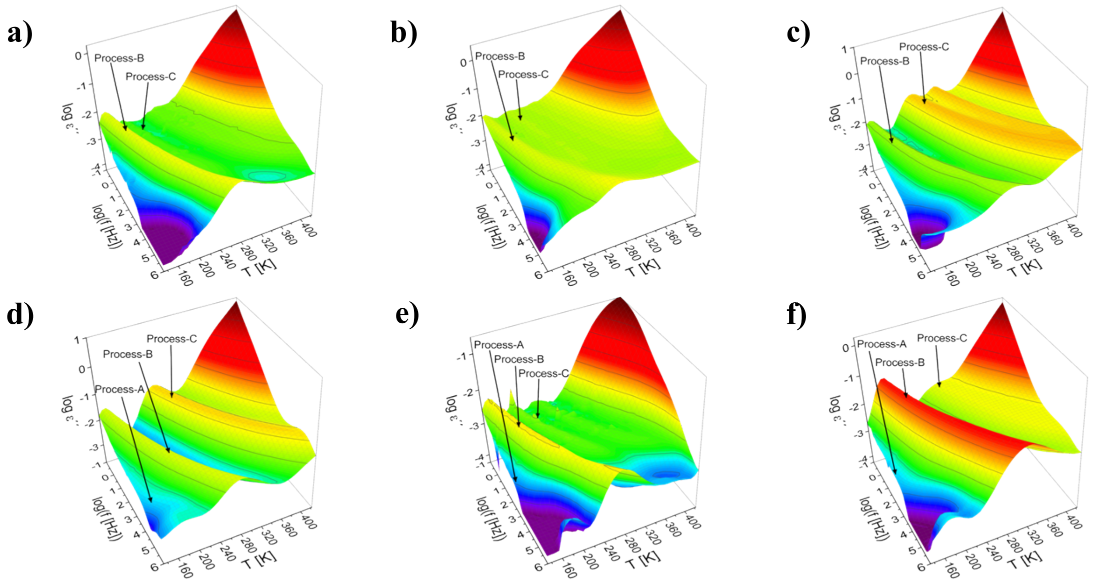

Figure S6. Dielectric loss ε'' as function of frequency and temperature as 3D representations during the heating cycle for a) Mg90Co-MOF-74, b) Mg70Co-MOF-74, c) Mg25Co-MOF-74, d) Mg90Ni-MOF-74, e) Mg70Ni-MOF-74 and f) Mg25Ni-MOF-74. Arrows indicate the dielectric processes.

Table S4. Estimated activation energies and prefactors for the probed processes

Name	Process-A		Process-B		Process-C	
	E_A [kJ/mol]	$\log(f_\infty[Hz])$	E_A [kJ/mol]	$\log(f_\infty[Hz])$	E_A [kJ/mol]	$\log(f_\infty[Hz])$
Mg100-MOF-74	-	-	41	12.8	54	13.6
Co100-MOF-74	-	-	39	13.3	63	15.3
Ni100-MOF-74	8	6.4	-	-	74	14.9
Mg90Co-MOF-74	-	-	42	13.8	49	12.9
Mg70Co-MOF-74	-	-	36	12.9	55	14.4
Mg50Co-MOF-74	-	-	37	13.1	55	12.5
Mg25Co-MOF-74	-	-	36	12.2	62	13.3
Mg90Ni-MOF-74	17	9.5	40	13.4	68	13.3
Mg70Ni-MOF-74	20	9.6	41	13.7	65	12.9
Mg50Ni-MOF-74	18	9.4	42	13.9	68	13.5
Mg25Ni-MOF-74	8	5.7	43	12.9	77	14.0

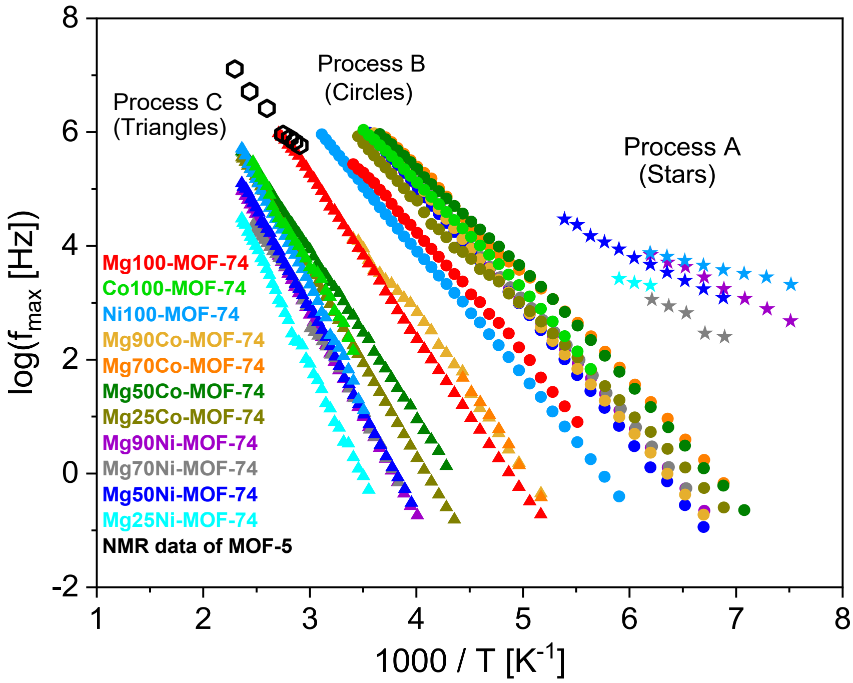

Figure S7. Relaxation map (Arrhenius diagram) of the MOF-74 samples. The different symbols symbolize the different processes as indicated. Every color represents different samples as it is indicated on the left bottom. Black hexagons denote the NMR data of MOF-5 taken from Gould et al. (J. Am. Chem. Soc. 2008, 130, 3246–3247).
